# Enhancing the ethical conduct of a longitudinal cluster-randomized trial of psychosocial stimulation intervention for children with complicated severe acute malnutrition through Rapid Ethical Assessment: a qualitative study

**DOI:** 10.1186/s12910-021-00578-7

**Published:** 2021-02-04

**Authors:** Tesfalem T. Tessema, Andamlak G. Alamdo, Eyoel B. Mekonnen, Fanna A. Debele, Juhar A. Bamud, Teklu G. Abessa, Tefera Belachew Lema

**Affiliations:** 1 Department of Public Health, St. Paul’s Hospital Millennium Medical College, Addis Ababa, Ethiopia; 2 Department of Maternal and Child Health, Silti Zone Health Office, Worabe, Ethiopia; 3 College of Education and Behavioral Sciences, Jimma University, Jimma, Ethiopia; 4 College of Public Health and Medical Sciences, Jimma University, Jimma, Ethiopia

**Keywords:** Rapid ethical assessment, Informed consent, Southern Ethiopia

## Abstract

**Background:**

Informed consent is a universally accepted precondition for scientific researches involving human participants. However, various factors influence the process of obtaining authentic informed consent, and researchers particularly working in resource-poor countries often face considerable difficulties in implementing the universally recommended procedures for obtaining informed consent. We have conducted this Rapid Ethical Assessment (REA) to accommodate the local cultural norms and to understand the relevant ethical issues in the Silti community before the conduct of a cluster-randomized controlled trial.

**Methods:**

This REA was conducted in two purposively selected Woredas/Districts and Worabe Town administration of Silti Zone. Data were collected using in-depth interviews and focus group discussions. Purposive and convenient sampling techniques were used to select respondents. Five in-depth interviews and 15 Focus Group Discussions were conducted in the Amharic language. The collected data was transcribed, translated, and analyzed using a thematic approach.

**Result:**

Most of the community members never heard about research and therapeutic misconception was common. In the area, the permission of people working in the formal and informal community administration is essential before approaching individuals. The male head of the household should also be involved in the decision before individual household members participate in research. Furthermore, sensitizing the community using public and religious gatherings was suggested before individual recruitment. In the consent process, delivering selected information particularly the purpose and benefits of the research was emphasized and the tendency of preferring verbal consent was documented despite the willingness of the individuals to sign on the consent form. Local health workers were identified as appropriate personnel to communicate information and the procedures of the research were found to be acceptable. However, the value of small incentives was suggested to motivate potential participants. Finally, involving all concerned stakeholders and respecting the cultural norm of the community was emphasized.

**Conclusion:**

Through REA, we understand the research awareness of the community, their expectation, and the cultural norms relevant to the ethical conduct of research. It enabled us to devise culturally sensitive and scientifically sound strategies to secure authentic informed consent. The process of conducting REA was found to be feasible, quick, and efficient.

## Background

Informed consent is a universally accepted precondition for scientific researches involving human participants based on internationally recognized and nationally adapted guidelines [1–4]. However, obtaining informed consent is influenced by a range of factors including the cultural setting in which the research is conducted, communication issues that affect comprehension of information, and the discrepancies in social and economic power [1]. As a result, researchers particularly working in resource-poor countries often face considerable difficulties in implementing the universally recommended procedures for obtaining informed consent [3]. In these settings, the lack of linguistic equivalents for some scientific terms including research, the higher rates of illiteracy, and the unfamiliarity of the community to the concept of research were some of the challenges in the application of informed consent [4, 7]. In Ethiopia, for example, 84.6% of health researchers and regulators reported a lack of satisfaction with the current informed consent process. This may be related to their evaluation where they thought that the consent process and the information given were not adequately understood by study participants and the best interests of study participants were not sufficiently considered. They also reported a lack of clarity and inadequacy of information, inappropriate use of language and terminologies, failure to consider cultural differences, power imbalances, coercion, and undue expectations of the community in the consent process of the Ethiopian medical research context [6].

In certain regions of the world, family members, religious and community leaders play an important role in the decision-making process for participation in medical research. For example, in Cameroon, consent for biomedical research should first be given by the fon (local traditional authority) [7]. In the context of rural Ethiopia, the involvement of significant other people such as family members, relatives, neighbors, community elders, and religious leaders is found to be important for any important decisions including participation in research [8–11]. Moreover, written signatures (thumbprint) may be difficult to obtain from potential research participants in some communities [8–10].

The robust body of work on the ethical conduct of scientific investigations highlights the application of general ethical principles of research is difficult to accomplish without the knowledge of the cultural context within which a study will take place [8, 12]. A study conducted in Ethiopia revealed that 95.4% of health researchers and regulators asserted the need to contextualize consent to the study setting and the need to conduct Rapid Ethical Assessment (REA) during the pre-test (pilot) phase of studies was suggested in settings where the ethical issues were not already mapped out [6].

REA is a brief qualitative intervention designed to map the ethical terrain of the research setting preferably before a research team starts recruiting participants to connect ethical principles to contexts and realities on the ground. It uses rapid ethnographic techniques to discover, describe and respond to the ethical issues specific to a particular research setting, and to help researchers to address the issues that genuinely matter to proposed study participants and their community [11]. Its methodology employs a constellation of action research, rapid assessment, and ethnography. The assessment is conducted among key community stakeholders and its findings can be utilized to inform and guide the research consent process; ranging from the conception and development of the consent form to the way consent is obtained [6, 12]. The tool was adapted and previously used in resource-poor countries [6–10, 13–15]. It was also found to be appropriate and feasible for the Ethiopian research setting and relevant and acceptable to the Ethiopian research community [11].

In previous studies, the REA identified important issues that guided the ethical conduct of different studies and improved the informed consent process in various settings. In the urban setting of Ethiopia, using short and understandable tools, and competent, compassionate, and respectful enumerators of the same gender were suggested. Alternative terminologies for some medical terms were also put forward to be used in the study [9]. Abay and associates noted low community awareness about health research and participant rights in Northern Ethiopia. Some medical procedures were also found to be highly embarrassing and verbal consent was preferred to written consent [14]. In another study conducted in Northern Ethiopia, respondents suggested sensitization meetings, visiting the households with community workers, providing trial information by someone with deep local knowledge, the use of local analogies to explain research terminologies, the value of small incentives, and the use of key community members to quell rumors arising in the course of the trial [5].

We have conducted this REA to accommodate the local cultural norms and to understand the relevant ethical issues in the Silti community, Southern Ethiopia before the conduct of a cluster-randomized controlled trial. Indeed, similar studies were conducted in different parts of Ethiopia employing REA [6–10, 13, 15]. However, in Ethiopia, a country with significant diversity in terms of cultural norms, religious ideologies, language, and social structure, the assumption on the existence of varying ethical concerns among different segments of the community could be relevant. It was also one of the reasons for the conduct of this REA in the Silti community, a Muslim community in Southern Ethiopia where the level of illiteracy is high, the community understanding of health research is unknown and the potential ethical issues were not mapped out to guide the ethical conduct of health researches. Furthermore, no similar longitudinal trial was ever conducted in the area before. The involvement of children who are a vulnerable segment of the community according to the Ethiopian National Research Ethics Review Guideline [2] and the long follow-up of the potential participants through home visits and the associated repeated measurements and contacts with different research team necessitated the conduct of the REA before the trial is to be carried out. We were particularly interested in identifying the potential ethical issues in the area to tailor the consent process to the local context before the initiation of our longitudinal research. The trial is aimed to examine the effect of psychosocial stimulation interventions provided with the routine in-patients care and for six months thereafter on the development, growth, and treatment outcome of severely malnourished children age 6–59 months in Silti Zone, of Southern Ethiopia. The detail of the trial is presented elsewhere [15].

## Methods

### Study area

Our longitudinal cluster-randomized trial is being conducted in Siliti Zone among all health facilities that have a well-established stabilization center for the provision of inpatient care for children with Complicated Severe Acute Malnutrition (SAM). Silti Zone comprises nine Woredas with a capital town named, Worabe, which is located 175 km south of Addis Ababa. According to the Ethiopian Central Statistics Authority, it has a total population of 937,212, with 837,207 (89.35%) people living in rural areas [16]. Islam is the predominant religion in the area. Silti Zone has 189 health posts, 35 health centers, four hospitals. Educational Attainment in Ethiopia is very low particularly among rural residents where 56.8% of women and 32.5% of men have no education [17]. This REA was conducted in three purposively selected locations namely: Hulbareg Woreda, Silti Woreda, and Worabe Town Administration which were selected in consultation with the staff of Zonal Health Office to represent the rural, semi-urban, and urban areas in Silti Zone.

### Data collection and participants

Data were collected using in-depth interviews (IDIs) and Focus Group Discussions (FGDs) from October- November 2019. Separate semi-structured IDI and FGD guides are used to facilitate interviews and discussions respectively. The guides were prepared based on the tool adapted to the Ethiopian context [12] and found to be feasible [11] and used in several studies, [6, 8, 10, 14, 15]. The interview and discussion guides developed for this study is provided as Additional file 1. The tool included questions on the socio-demographic issues, about the formal and informal structure of the community, their understanding and familiarity with research, the methods to approach the community and the key people and organizations to be involved in the process, the decision-making practices of the community and who should decide for research participation at the community, household and individual level, about the communication methods in the consent process, the acceptability of the planned research procedures and the potential barriers to participation. Experts^1^ were also asked about their experience and the existing challenges in the consent process and their recommendation to improve the process. The questions included probes designed to reveal greater details to explore specific aspects of an issue when necessary. The research team translated the tool into Amharic (the local language) and given to an experienced public health expert who is a native speaker to back-translate into English to ensure the accuracy of the translated version. Non-probability purposive and convenient sampling techniques were used to select IDI and FGD respondents respectively. Five FGDs lasting about one to one-and-a-half-hours were conducted with a group of 6–10 individuals with varying age groups: (1) mothers of children with SAM; (2) fathers of children with SAM; (3) mothers of healthy children; (4) fathers of healthy children; (5) with a mixed group of women and men community members. Fifteen one-on-one IDIs lasting for about 30 to 45 min were conducted with two Health Extension Workers (HEWs)^2^, two health service providers, two health facility managers, two Woreda/District health office managers, and two Kebele^3^ managers, one community, and two religious leaders, and two public health researchers from Worabe University. The staff of the Woreda health departments, the health facilities, the research office of Worabe University and the kebele offices assisted the research team in identifying eligible respondents representing different stakeholders. Health service providers working in the nutrition unit assisted in the identification of parents whose children were admitted to the nutrition unit with SAM while HEWs identified parents of healthy children and other respondents from the community. From the community, adults (age > 18) and permanent residents and from among experts those with more than 2 years of managerial, clinical or research experience were recruited. The research team contacted the potential participants, explained about the study and invites them to participate in the study. None of the approached IDI and FGD participants refused to participate in the study. The actual number of IDIs and FGDs were decided based on the data saturation, which was considered when no new information was obtained from interviews or discussions that were analyzed in the course of data collection.

Data were collected in the private areas such as the offices in the health facilities, Woreda Health department, and Kebele where the respondents and the research team feels privacy and no one was present in addition to the respondents and the research team. All interviews and discussions were conducted in Amharic and tape-recorded and field notes were also captured in the process. An experienced team of researchers from the Cluster Randomized Trial of psychosocial stimulation intervention for children with SAM (EPSoSAMC Trial) who have considerable experience working in the community conducted the REA. A relaxed interviewing and discussions were used encouraging participants to talk openly about their views and interact with each other. Throughout this paper, the Consolidated Criteria for Reporting Qualitative Research (COREQ) was followed [19].

### Data management and analysis

The preliminary analysis was initiated alongside the data collection. Before the actual analysis, the data collected in a digital recorder was transcribed and translated from Amharic into English. A thematic analysis was used to analyze the data. To ensure the rigor of this study, three experienced qualitative researchers analyzed the data in-group. They repeatedly read transcripts and assign codes after discussions to clarify an individual’s codes and their meanings. The codes are then organized into categories by looking at connections between the identified codes. Finally, the different categories were linked to developing themes based on the objectives of the study. The Ethiopian National Research Ethics Review Guideline requires respondent validation of the researcher’s analysis before dissemination of the qualitative research findings [2]. Therefore, the draft finding of the study was shared with selected respondents to ensure the accuracy of the reporting. Based on the analysis five themes were developed and presented accordingly.

## Result

### The socio-demographic characteristics of the study participants

In total 58 (Female 25 and Male 33) respondents took part in the study. From the IDI respondents, 11 were male and three had no formal education. From those who had formal education, three, five, and three completed certificate, BSc degree, and MSc degree level training respectively. Regarding the profession of the educated IDI respondents, two were Nutritionists, four were nurses, two were public health officers and three were HEWs. The age range of the IDI respondents was 28–75 years. Among the FGD respondents, 21 were female, 41 were married, all were Muslim and 27 had no formal education. Nearly all [19] of the women FGD respondents were housewives while a similar proportion [21] of the men respondents were farmers. The age range of the FGD respondents was 19–57 and they have 1–9 children at the time of data collection (Table 1).

**Table 1:** The characteristics of the REA respondents, Silti Zone, Southern Ethiopia, 2019

	Number
Characteristics of FGD Respondents (n = 43)	
Sex	
Women	21
Men	22
Marital status	
Married	41
Other	2
Religion	
Muslim	43
Educational status (Women)	
No formal education	15
Primary education	6
Educational status (Men)	
No formal education	12
Primary education	7
Secondary education	3
Occupation (Women)	
Housewife	19
Others	2
Occupation (Men)	
Farmer	21
Others	1
Age	
Range	19–57
Number of children	
Range	1–9
Characteristics of IDI Respondents (n = 15)	
Sex	
Male	11
Female	4
Age	
Range	28–75
Educational status	
No formal education	3
Secondary education	1
Certificate	3
BSc	5
MSc	3
Profession (n = 11)	
Nurse	4
Public health officers	2
Nutritionists	2
HEW	3

### About the community and their understanding of research

A good understanding of the formal and informal structure of the community is essential for the successful conduct of any community-based study. The REA identified Kebele managers, religious, and community leaders as influential people having a meaningful role for the smooth conduct of research activities in the area. A Kebele manager is a trusted individual selected from the community who is responsible for the overall administrative works in a given Kebele. Religious leaders are respected individuals representing a specific religious group while community leaders are usually elderly people. We recognized the value the community often gives to these people where their prior permission is important for the conduct of any community-based activities including research. The respondents also mentioned the importance of involving HEWs considering their close working relationship with the community.

Regarding the prior research projects, the experts mentioned that very limited community-based studies (usually small-scale research projects) were conducted in areas declaring that no longitudinal study as the planned research work was conducted.

Very few cross-sectional studies were conducted in Silti Zone… research with long follow up is never conducted in this area. (IDI, Male, Facility Manager).

SIlti Zone is established 10 years ago and the university is still new. We do not have adequate human, financial, and other key resources. As a result, limited studies were conducted in our community. (IDI, Male, Researcher).

Considering the scarce research works conducted in the area and the illiteracy level of the community, the experts repeatedly mentioned that the community might have an inadequate understanding of the research.

Unfortunately, our community is illiterate and they are not well exposed to researches before. Therefore, they may not understand the research or misapprehend it with medical intervention and other benefits. (IDI, Male, Service Provider).

If you go to Jimma (a town in Southwest Ethiopia where Jimma University has been operating for decades), the community could tell you better about research. It is because of their frequent exposure and you should not be surprised if our community has poor understanding. (IDI, Male, Researcher).

The community members further elucidated the response of the experts. Most of them never heard about “Temeramirot” (the local terminology for research). While others mentioned research equating to medical care, planning, and doing beneficial interventions such as the construction of health facilities and latrines and helping poor families with financial and other benefits.

…. I do not know about research, never heard about it and it is your role either, the role of the educated ones. (IDI, Male, Community Leader)

Some organizations came to our community to help children from poor families. They were doing research. (IDI, Male, Religious Leader).

… It is about planning. For example, HEWs sometimes came here to discuss some important issues with us. Then we plan with her what and how we should do things. It is research … (FGD, Female, the Mother).

### The method approaching the community

In the study area, the tendency of approaching potential participants directly is nearly impossible. Involving peoples in the formal and informal chain of the community administration and local health workers particularly HEWs was proposed as a suitable strategy before approaching potential participants.

To successfully conduct the research, you have to involve all concerned individuals including the Kebele, religious, and community leaders and health workers before contacting the study subjects. (IDI, Male, Facility Manager).

Most experts underlined the importance of sensitizing the community before individual recruitment. Two approaches were suggested: the first is to call for public gathering through the Kebele managers and conducting sensitization with the local health *workers* and HEWs.

… The good thing is to provide detailed information to the community with our health workers and HEWs as they are well-trusted in the community. (IDI, Male, Woreda Health Office Manager).

The community should be told about your work. It is easy; the Kebele Manager can call the meeting. (IDI, Male, Community Leader).

The second approach is using the major religious gatherings that have been regularly happening every Friday (Jumma/Muslim Prayer Day) at Mosque settings, which are available at each Kebeles.

It is very nice if you sensitize the community in the Mosque particularly on Jumma (Friday Prayer of the Muslim community) as all people go to Mosque for prayer …. (IDI, Female, HEW).

… Our people should have the information first. We could talk about your work in religious gatherings as far as it is not against our Sheria (Islamic law). (IDI, Male, Religious Leader).

### Recognizing the role of others in the decision to participate in the research

Different opinions were offered on who should decide at the level of community and for individual household members to participate in research. At the community level, the permission of people working in the formal and informal community administration is essential.

First, you need to have the approval of our leaders including the kebele managers, religious, and community leaders. Without their recognition, you will not be successful. (IDI, Female, HEW)

… The good understanding and the favorable views of the community leaders will have a valuable contribution to effective individual recruitment. (IDI, Male, Manager).

For the research participation of the individual household members, most of the respondents said that the male head should make the decision. They stressed that women should also seek the approval of her husband before making decisions.

In this community, the male head of the household is making a major decision including for research participation of individual household members. (IDI, Male, Researcher).

To participate in research, even a woman should consult her husband before making a decision. (IDI, Male, Service Provider).

Despite the favorable views on the dominant role of the man as a household head, few women respondents mentioned that both women and men members of the household discuss and make the decision together. However, experts with substantial experience working in the community emphasized the importance of including the men head of the household in the decision considering the nature of the planned research that requires long-term follow-up, repeated home visits, and the involvement of children and other family members.

In your research, if you do not involve the husband in the decision, you might be stuck in the middle when he (referring to men household head) becomes aware that the wife decided without his knowledge. (IDI, Female, HEW).

He (referring to men household head) is the one to decide for the household members. Once he made the decision, he can freely allow you to continue doing your stuff with the mother and the child. (FGD, Female, Community Member).

When asked who should decide on behalf of children to participate in research, nearly all of the women community members also stated that they need the approval of their husbands before the decision.

I will not decide alone… The father should also decide. (FGD, Female, Mother)

If you approach me to include my child, I will tell you that I need to consult my husband first. It is after his approval that I will allow you to include my child. (FGD, Female, Mother)

It has been also argued by few respondents mentioning the influence of the husband’s mother in low on the household decision-making process. It was understood from the REA that, the husband’s mother in low usually lives in the household if they are alone and that she has an important influence in the household decision.

The influence of the husband’s mother in law is also important in any decision made at the level of the household. (IDI, Male, Manager).

### Communicating information to potential participants in the consent process

Given the existing situation of the study subject and the community, all experts agreed that the consent process should be adapted to the local setting. However, different views were offered regarding the content of the information that should be communicated in the consent process. Considering the nature of the planned research and other important issues, most of the experts emphasized the delivery of the most important information. Among the recommended contents of the recognized ethical guidelines, the purpose and benefits of the research were repeatedly mentioned as the most relevant information to be communicated to the potential participants.

Since the planned research involves minimal risk, you should only give important information such as what you are out for, the purpose and benefits of the research. (IDI, Male, Researcher).

You are going to work with the illiterate community who may have never participated in research before. I believe that you should focus on giving the most important information. (IDI, Male, Service Provider).

However, few interviewees argued against providing limited information mentioning the following:

I think all information should be given to the study subjects in the simplest form possible. … If you invest your time to explain things, I am sure anyone can understand… even the illiterate community members. (IDI, Male, Service Provider).

When it comes to the delivery of the information in the consent process (giving written or verbal information), all the respondents agreed that the information must be given verbally as most of the potential participants lack the ability to read and understand the contents of the document.

… Most of our community members could not read and understand the information in the written documents. How could you give them written information? You must read the content of the document verbally. (IDI, Female, HEW).

To give information to the potential participants, the experts repeatedly suggested the inclusion of the local health workers into the research team underscoring the trust that the community has in them, their close working relationship with the community, and their good understanding of the local issues.

For your case, consider including health workers working in the local health facilities in your team. They could provide the information to the study subjects who could easily trust them. (IDI, Male, Manager).

Regarding the willingness of potential participants to sign the consent form, all of the participants agreed that having the written signature could not be a problem in the community.

Signing on the document is nothing. What we matter most is our word. That is all. If you want our signature, we can sign, that is easy. We cannot change our words; it is what we matter… (FGD, Male, Farmer).

The researchers with considerable work experience in the area, however, underlines the importance of telling the detailed information and creating a trusting relationship before requesting individuals to sign on documents.

Our community is very flexible and they have a long-existing trusting relationship. I am sure they do not have any problem with signing as far as they trust you and understood the process. (IDIs, Male, Provider).

### The acceptance of the planned research procedures

We explained the procedures of the planned research that was presented elsewhere [15] and asked the respondents whether the procedures are acceptable to the mothers. They all agreed that the procedures would be acceptable as far as they understood its purpose and benefit to the community.

Are you asking our willingness if you follow the health of our children and coming home to see them? Of course, we will be happy … (FGD, Female, Mother).

They will be very happy if you visited their home. It will also improve their trust … (IDI, Female, Kebele Manager).

I know the community very well. Mothers do not have any problem if you go to their home and ask them to bring their child once they understood your work… (IDI, Female, 32 years, HEW).

To motivate mothers and achieve effective retention, experts suggested the value of small incentives to compensate the time the busy mothers dedicate to the research activities. They identified soap, iodized salt, play materials, child clothes, and monetary rewards as simple incentives.

I think mothers should be motivated for their engagement. If you give them small incentives, they will happily participate in your research. (IDI, Male, Manager).

The HEWs put forward a contradicting opinion mentioning that:

I am working with our mothers… I am sure they will expect nothing because of their participation. It is the community leaders and the community works who may expect incentives… (IDI, Female, HEW).

However, the mothers who participated in the FGDs suggested the importance of considering community-based events such as the market and prayer days.

We have to go to the market on Market days and Friday is our Prayer day where we should pray at Mosque. You should not appoint us in these days. (FGD, Female, Mother).

A HEW who has been working in the area for more than nine years further clarified the issue mentioning:

….it is good if you are not scheduling activities in market days… As a Muslim community, Friday is also a busy day here… (IDI, Female, HEW).

### Potential barriers to participation

A few experts suggested the importance of considering the current political situation of the country where the community could easily be suspicious of the outsiders and that wrong rumors could be easily disseminated with the potential to discourage participation. They underlined the value of working with all concerned individuals and organizations to gain the trust of the community and easily manage any problems that may arise in the conduct of the planned research.

You know the current politics… As an outsider, you should have a good working relationship with everybody to ensure the smooth conduct of your research and to manage any unforeseen problems. (IDI, Female, Kebele Leader).

Nearly all of the respondents suggested the importance of respecting the cultural norm of the community. One major issue repeatedly mentioned was the tendency to influence the religious ideology of the community.

We all are very serious in our religion. … Some time ago, one organization (name mentioned), came here to support poor families. Letter on, we discovered that it has a hidden religious agenda that made us very nervous… (IDI, Male, Religious Leader).

As far as it (the research) is not against our Sheria (Islamic law), and if you respect our culture and religious practice, we will support you. (IDI, Male, Religious Leader).

## Discussion

We conducted the present REA to understand the potential ethical challenges relevant to the study area and to develop a tailored consent process before the conduct of a large-scale longitudinal study. A good understanding of the basic concept of research among people approached is essential to secure authentic consent. The REA found out that the community in SIlti Zone has inadequate understanding of the concept of research. In studies conducted in Southern and Northern Ethiopia, a similar finding was noted [9, 14, 15]. Ethiopian health researchers and regulators also shared the insights that the community’s awareness about research and research ethics elements are not well developed [6, 21]. These may have led to the therapeutic misconception of health research, the belief that participation in research will result in some medical benefits to the participants [21]. The misconceptions of health research with medical interventions, aids, and other immediate benefits were reported in many studies conducted in resource-poor countries [6, 10, 14, 15, 23]. We found a similar finding where most of the community members understood research equating it to medical care while others related research with planning and doing some beneficial interventions.

In line with similar studies, our study demonstrated that the low level of societal literacy and their limited familiarity with the research works was among the major reason for the inadequate level of research understanding [13]. Indeed, education is one of the most important aspects of social and economic development. However, educational attainment in Ethiopia is generally low especially among rural residents where 54% of females and 39% of males have no education. In Southern Ethiopia where our study is being conducted, only 1.7% of females and 2.2% of males completed primary education [17]. Nevertheless, some level of understanding regarding the concept of research among inadequately educated participants who had familiarity with health research was noted [10, 24]. In a study conducted among people who had mostly no formal education, the difference in the research understanding among those who have experienced agricultural studies with those who had no experience was noted [14]. Some researchers working with illiterate communities argued against the notion that illiteracy is a barrier to research understanding and comprehension except for the understanding of abstract scientific concepts [24]. Bhutta also put a favorable argument advising to never consider illiteracy to mean that a potential participant is unable to comprehend complex information suggesting the value of presenting the information differently for these populations [3]. These findings suggest that the lack of experience and familiarity with research work could have a pivotal role in shaping the research understanding of the given community. In consideration of the fact that illiteracy is widespread in most resource-poor countries particularly in the poorest areas, devising an appropriate mechanism that assists illiterate people to have a basic understanding of research before the consent process could be important.

The tendency to approach the individual study subjects directly without considering the existing community structure may be difficult in some communities as individuals might lack trust in the outsiders and become reluctant to participate. In Silti Zone, it was found out to be important to involve and get the permission of key and influential peoples such as the kebele managers, and religious and community leaders who have a meaningful role in the formal and informal community-based administration before approaching individuals. The role these people may have and their level of influence might vary based on the culture, religion, and the structure of the community in a given area. For example, in Cameroon, the fon (local traditional authority) plays a very important role as the entry point to the community [23]. In South-Eastern Tigray, religious leaders were the most accepted people to reach the community [14]. While in East Gojjam, kebele leaders and law enforcement were identified as the keys to approaching the community [5]. Considering the value of respecting the community and creating a good relationship, seeking the involvement of these people before reaching the potential study subjects could have a meaningful place for the smooth conduct of the planned research.

Conducting community sensitization and giving collective information through public and religious gatherings was also suggested before approaching the study subjects. Using Mosques for community sensitization, which was specific to our study, was meaningful given that nearly all of the community members follow Muslim Religion in the study area. Other studies also recognized the value of community sensitization and group information provision using community gatherings such as churches and market places [14, 15, 24]. The REA threw valuable light to using the community sensitization and individual consent process as an opportunity to discuss the concepts of research with the community, which could improve their awareness and address the existing misunderstanding. The benefit of community sensitization in educating the community about research, enhancing the level of individuals' comprehension and clear misunderstandings was well documented [10, 15, 23].

The social relationships of an individual within families, institutions, and communities may influence the assumption of informed consent for a self-determined choice of individual study subjects [21, 26]. Therefore, the understanding of the decision-making tradition in a given community is important to devising appropriate strategies that balance respecting the individual autonomy for a self-determined choice against respecting the community-based decision-making tradition. In the study area, the permission of key and influential community members such as Kebele managers, religious and community leaders who are working in various hierarchies of the formal and informal community-based administration is essential. It is a common phenomenon in some societies in resource-poor countries where it would be culturally inappropriate to ask individuals for research participation without consulting the community or getting permission from community leaders [21]. In the context of rural Ethiopia for example, any important decision has to involve significant other people including community elders and religious leaders [5]. Given the intimate and shared living arrangement in these settings, recognizing the value of involving all concerned bodies before initiating any community-based activities including research work may be necessary. Based on the international guidelines that suggested the significance of accommodating the cultural context of a given population, the permission requirement of these people before approaching potential individual participants could be ethically sound as far as it does not compromise the requirement for an individual’s voluntary informed consent.

At the household level, a man has a dominant role in decision making on the behalf of other family members including the women to participate in research by culturally accepted status as the head of the family. A similar finding was reported in Cameroon where most women even advocated for male responsibility in giving consent for research participation [23] and in some areas of Uganda as well [21]. This is particularly relevant in Muslim communities where the permission of the husbands must be sought and obtained before accessing women for surveys [26]. Furthermore, it was rarely mentioned that the mothers in law who usually lives with her son when she is alone has also an influence on the household level decision including for research participation of individual members. In South Asian regions, the mothers-in-law commonly exert power over daughters-in-law to the extent that the women may not be able to express personal opinions on even minor household matters including research participation [21]. In such circumstances, the decision-making status of women could in part be the possible barrier to voluntary participation in research [9]. The situation where another person including the male family head customarily has the authority to make decisions on behalf of others on whether they will participate in research is challenging considering the recommendation of the universal ethical standard of informed consent. However, attitudes have been changing dramatically in much of Africa, where many women, especially in non-Muslim societies, are now cultivating a more assertive position concerning healthcare [21]. For example, in a study conducted in Northern Ethiopia, women reported their individualized decision-making role in any aspect of their life including for research participation [14]. Until it becomes practical in similar settings, researchers, therefore, need to take time to work with all concerned bodies at the various stage of the research and take culturally sensitive measures to secure authentic informed consent from women participants to the greatest extent possible. Despite this, considering the nature of the planned research that requires long-term follow-up, repeated home visits, and family involvement, a sound argument to involve the male head of the household was proposed.

The ethical procedures that are designed based on the specific attributes of the population in high-income countries may be ineffective for implementation in some resource-poor countries because of the significant differences in cultural and social environments [21]. Considering this, the Ethiopian Research Ethics Review Guideline suggested that the content of the information sheet should consider the local culture and values, as well as the level of understanding and competence of the research participant [2]. About 95.4% of Ethiopian researchers, regulators, and IRB members also thought that the internationally recognized consent process and the elements of information sheet are often inappropriate in some societies demanding contextualization to the given setting [6]. A similar finding was reported in Northern Ethiopia [5]. In this REA, similar views were shared mentioning that the consent process should be adapted to the local setting considering the existing situation of the study subject, the community, and the cultural issues.

The requirements for informed consent assume that individuals are autonomous agents with the capacity for expressing a self-determined choice after information on the purpose, risks, benefits, and alternatives are thoroughly described and understood [25]. However, various challenges were reported in the provision of information to the potential participants in different settings. In a study conducted in Ethiopian universities and health research centers, consent-related problems including the provision of inadequate information, lack of clarity of the information, the use of inappropriate language, and terminologies were documented [6]. In other studies, the tendency of stressing more on information related to the potential benefit and de-emphasizing information on the potential risks of participation was noted [8, 14, 15, 24, 28]. The reluctance of researchers to provide all the relevant information in a way that is understandable and appropriate, in particular disclosing the risk related information might be intentional due in part to the fear that it might frighten the study subjects with the potential to deter participation [28]. Moreover, the frustration of the researchers regarding the lengthy and complex disclosure requirements for informed consent and the tendency of focusing more on the consent than on informed consent, which is based on prior information and comprehension was also noted [6, 26]. Our study found similarly finding where most of the experts often expressed the difficulties of communicating information based on the recognized ethical requirement in some communities. The consideration of the existing local situations of the community such as the low level of literacy and the lack of familiarity with the concept and procedure of research that possibly influences the comprehension level of the potential participants was suggested. The nature of the planned research where it was assumed to have minimal risk was also mentioned as a favorable argument. Yet, a meaningful argument was shared underscoring that the researchers usually focus on recruiting as many as possible study subjects and getting the required data quickly than giving complete information that is relatively time-intensive. It was also suggested that even illiterate subjects could understand the information as far as it is customized for the level of their understanding and literacy level of a given population. This concern is consistent with the universal ethical standard of informed consent that requires the information provided to research participants should be analyzed in terms of containing the basic elements of information which should be communicated accurately and in an understandable and appropriate way [1, 3, 4]. In communities where there is a low level of literacy, it is common to see the tendency of preferring oral consent to written consent (8–10, 24). The study subjects may also hesitate to sign (using thumbprints) on the consent form although they agreed to take part in the study. For example, in Ethiopia, several studies reported the difficulty of obtaining the written signature [8, 10]. The commonest reason appeared to be the fear of legal issues and accountabilities associated with signing on the document. The past experiences of the study subjects such as losing the land by signing papers and the fear of any other adverse repercussions such as stigmatization could have made people suspicious of signing documents [22, 26]. In contrary to most of the studies, it was worth noting that, signing on documents including for research participation is not a problem in the Silti community, although we noted the preference for the information to be communicated orally. In explaining this, the flexible nature of the community, their frequent experience in signing on documents, and the long-existing trusting relationship were repeatedly mentioned.

After explaining the procedures of the planned research (the detail was presented elsewhere) [15], the procedure was found to be acceptable for the potential study subjects. However, the importance of considering community-based events such as the market and the prayer days of the community was suggested for consideration while scheduling activities with the potential study subjects. In the REA conducted in Addis Ababa, participants confirmed as there is no problem with repeated interviews, but emphasized the importance of making an allowance for the home and outdoor responsibilities of the women in deciding the timing of the interview [9]. The value of small incentives to motivate the potential study subjects was also suggested to ensure effective participation. The pivotal role of incentives to achieve effective recruitment and retention was also recognized in similar studies [8, 10, 15]. International ethical guidelines also recognized the value of incentive as far as it is large or extensive enough to induce study subjects to consent to participate against their better judgment [29]. In the light of the local circumstances, the incentives proposed by the respondents could be appropriate to motivate the potential study subjects without causing the significant ethical concerns of influencing the individuals' informed decision to take part in the research. In our view, the finding regarding the incentive expectation of community leaders and community works for their engagement in the community mobilization efforts which was also reported in similar studies was also meaningful [9, 27].

The REA identified some of the potential barriers that could deter the study subjects from taking part in the planned research. The suspicious view of the community to the outsiders and the associated wrong rumors that may easily arise and disseminate in the course of the research was mentioned perilous with the possible potential to influence participation. Similar studies conducted in different parts of Ethiopia also reported the distrust of the community to the newcomers and the potential of false rumors in creating misconceptions in the community [8, 9]. The tendency of ethnic bigotry and the existing wave of insecurity in Ethiopia contributed to the mentioned phenomena [30]. In line with the finding of similar studies, garnering the support of all concerned stakeholders, a prior community sensitization to disseminate the right information, and ensuring a clear understanding of the nature of the research was suggested [8, 31]. Respect for cultural norms builds a foundation of trust between researchers, study subjects, and the local community [25]. The value of respecting the cultural norms of the community including the religious ideologies and practices were also repeatedly suggested, as the lack thereof potentially dissatisfy the study subjects and become the barrier to participation. In similar studies, the understanding of the culture, religion, and livelihood of the people approached and the circumstance of the study subjects was mentioned as essential [10, 15]. Therefore, we were cautious in selecting the play activities and materials, in selecting home visitors who were recruited from the local community with a good understanding of cultural norms and religious issues of the community including the dressing codes. Due attention was also given to the interaction happening with mothers/fathers with an opposite-sex research team.

To mention some of the limitations, we used the referral of local people in the selection of respondents. For example, at the community level, FGD discussants were selected by HEWs while at the health service providers selected mothers/fathers of children with SAM. Similarly, health facility managers, Kebele managers, and the Research Office head of Worabe University selected the health workers, community-level IDI respondents, and researchers respectively. Consequently, we are not quite certain whether the respondents selected for the study actuality represents the variability of the community and the selection process is free from the potential selection bias. The study was conducted in the Silti community, a Muslime community in Southern Ethiopia and our findings may not fit the realities of other settings given the remarkable diversity across regions, religions, and ethnicities of Ethiopia.

## Conclusion

The REA enabled the understanding of the community, their research awareness, expectation, and the cultural norms relevant to the scientific ethical standards. It facilitated the identification of the potential ethical issues that may arise in the preparation and conduct of the planned longitudinal research. In the meanwhile, it informed the team to devise culturally sensitive and scientifically sound strategies to secure authentic informed consent. Given the valuable output of the REA in improving the ethical conduct of relatively resource-intensive longitudinal studies, we thought that the process of conducting it is feasible particularly for researchers who have experience in conducting qualitative studies. As the name implies it is quick as it can be completed in only several weeks and it is efficient, as it does not require a huge investment. Plainly, the assessment is helpful to map out the potential ethical issues in the area and informed their solution for use in the conduct of the planned longitudinal cluster randomized trial.

## Supplementary information

**Additional file 1.** Interview and discussion guides.

## Data Availability

The datasets used and/or analyzed during the current study are available from the corresponding author on reasonable request.

## Abbreviations

COREQ: Consolidated Criteria for Reporting Qualitative Research
FGDs: Focus Group Discussions
HEWs: Health Extension Workers
Dis: In-depth interviews
IRB: Institutional Review Board
REA: Rapid Ethical Assessment
SAM: Severe Acute Malnutrition

## Footnotes

1 Experts included researchers, health providers, health facility and Woreda health office managers.

2 HEWs are the community workers managing health posts, which are the lowest primary health care delivery units in the Ethiopian health care structure.

3 Kebele represents the lowest administrative unit in Ethiopia.
