# Supplementary material for: Enhancing the ethical conduct of a longitudinal cluster-randomized trial of psychosocial stimulation intervention for children with complicated severe acute malnutrition through Rapid Ethical Assessment: a qualitative study
Source: BMC Med Ethics. 2021 Feb 4;22:10. doi: 10.1186/s12910-021-00578-7 (PMC7863315; doi:10.1186/s12910-021-00578-7)
Supplement: Supplementary file 1 — Additional file 1. Interview and discussion guides. [file 12910_2021_578_MOESM1_ESM.docx]

# **IDIs guide questions for managers, health and community workers**

**Personal questions**

- Can you please tell me a bit about your background and training?

**Prompt**: how long have you been working with malnourished children?

- How do you help malnourished children and their families?

**Prompt**: Providing health education, managing at the health facilities, following them after discharge, and how do you feel about working with malnourished children

**The nature of our study**

- How does the community describe malnutrition?

**Prompt:** cause, treatment, and prevention?

- How do people react to a malnourished child? The family?
- How do people in SNNPR or the study area understand childcare?
- How do people in SNNPR or the study area understand the importance of play to the child?
- How do people in SNNPR or the study area understand child growth and development?
- How do you think we should explain the association between play and the child's growth and development?
- How do you think the importance of play to the child is explained to participants?
- Is there anything that we haven’t raised about the nature of our study?

**Research conduct and consent process**

- How do you describe the research related literacy of the community?

**Prompt**: the level of exposure of the community to research activities

- How would you describe the understanding of research by the community?
- Do they understand the difference between research versus treatment or research versus aid?
- What positive and negative myths exist about research? If there is any, tell us the detail and about factors that contributed to it?
- How can their understanding be promoted? How do we address the misconceptions?
  - Have you asked participants from this area to consent to research in the past? If yes,
    - How well do you think this community understands the idea of the research and consent process?
    - How did you inform participants about the study?
    - What steps did you follow to approach and recruit participants?
    - How did you assess voluntariness?
- Are there any issues particular to this community in terms of getting consent? If, yes,
  - Have you experienced these problems? How did you overcome the problems?
- Why do you think people in the community participate in research?
- How easily participants understand our interest in our research?
- How do you think information to be told to patients and families?
  - **Prompt**:
    - Through the involvement of a trusted member of the community (e.g., Health facility staff, HEWs, HDAs)
    - Through the involvement of close families
    - Telling prospective participants confidentiality and anonymity will be maintained
    - Through dialogue between the community and the researchers
- In your opinion, what are some of the obstacles that hinder prospective participants from disclosing information to other people?
- What strategies did you use in the past to publicize the research findings? **Prompt**: community sensitization or other means
- What information participants would like to receive before they take part in the research?
  - **Prompt**: What the study is about? Who the researchers are? Why the study is conducted? Importance of the study to them or the community? Concerns of being involved in the study (e.g., confidentiality and the result will not be used to trace either participants or their family in any way), etc?
- How did you provide information about the study to participants?
  - Verbal or written
  - Group or individual information provision
- Do you think conducting longitudinal research with repeated measurements and household visits will pose specific issues?
- What would be some of the possible factors affecting the recruitment of participants to our proposed study?

**Decision-making norm**

- How do you describe the decision-making norm in this community?

**Prompt:**

- - How the families in this area usually make a decision about whether or not to take part in research? **Prompt**: Would the decision be made as a group or would one person make the final decision?
  - Do you think a family member should be consulted before an individual is making a decision to take part in research? **Prompt**: who is the one who should be consulted before making the final decision?
  - Do you think other members of the community should be consulted before making a decision to take part in research? **Prompt**: elderly, religious leaders, kebele leaders.
  - Which member of the community could make an individualized decision to take part in research? **Prompt**: adult male, adult women, male greater than 18 years, female greater than 18 years
  - Who should decide for small children (less than 5 years) to take part in research? **Prompt**: adult male, adult women, male greater than 18 years, female greater than 18 years
  - How do you describe a situation where the research participants are required to bring their child to the health facilities for the purpose of the research?
  - How do you describe a situation where the research team visits the research participants’ home repeatedly to give education, to ask several questions, and to play with a child included in the study?
  - Do you think other community members should accompany the research team during home visits? Who do you think is the appropriate member of the community? **Prompt**: HEWs, HDAs,
- Is there anything that we haven’t raised about the decision-making norm in this community?

**Conclusion**

- Where would we seek guidance if problems occur when conducting our study?
  - **Prompt**: communicate with kebele heads, IRB members, colleagues, refer to national ethics guidelines
- Where would we seek guidance to settle rumors that will arise when conducting our study?
  - **Prompt**: communicate with kebele heads, IRB members, colleagues, refer to national ethics guidelines

# **IDIs guide for researchers of Worabe University**

**Personal information**

- Can you please tell me about yourself?

**Prompt**: training (educational background, specialization), experience (research, ethics, fieldwork)

- How long did you stay working in Silti Zone?

**The community**

- Have you conducted any research involving the community in Silti Zone?
- How do you describe the research related literacy of the community?

**Prompt**: the level of exposure of the community to research activities

- Do you think research conducted in these areas raises different issues compared to the same research being carried out elsewhere in Ethiopia?

**Prompt**: cultural and diversity issues, language difference, literacy, poverty, cost-benefit analysis, social representations (e.g., age, gender, status…)

**Consent process**

- How do you think the consent forms and information sheets be prepared?

**Prompt**: aligned with the local people interest, language, educational status …?

- Tell us about some of the consent-related problems in conducting scientific investigations

**Prompt**:

- - Lack of clarity, inadequate information, language barriers, cultural differences, undue expectations, and power imbalances
- How do you describe the consent process from the research participants’ point of view?

**Prompt**:

- - Do you think that the study participants adequately understood the consent process and information given?
  - Do you think that the study participants were satisfied with the current consent process?
  - Do you think the best interests of study participants were not adequately considered with the current consent process?
- Do you think that consent should be contextualized to the study setting?

**Prompt**:

- - Considering the language, literacy level, cultural issues, and decision-making power of the community
- Is there anything that we haven’t raised about consent processes?

**The nature of our study**

- How do people in Ethiopia, in SNNPR, or the study area understand childcare?
- How do people in Ethiopia, in SNNPR, or the study area understand the importance of play to the child?
- How do people in Ethiopia, in SNNPR, or the study area understand child growth and development?
- How do you think we should explain the association between play and child growth and development?
- How do you think the importance of play to the child is explained to participants?
- Is there anything that we haven’t raised about the nature of our study?

**Decision making-norm**

- How do you describe the decision-making norm in this community?

**Prompt:**

- - How are families in this area usually make a decision about whether or not to take part in research? **Prompt**: Would the decision be made as a group or would one person make the final decision?
  - Do you think a family member should be consulted before an individual is making a decision to take part in research? **Prompt**: who is the one who should be consulted before making the final decision?
  - Do you think other members of the community should be consulted before making a decision to take part in research? **Prompt**: elderly, religious leaders, kebele leaders.
  - Which member of the community could make an individualized decision to take part in research? **Prompt**: adult male, adult women, male greater than 18 years, female greater than 18 years
  - Who should decide for small children (less than 5 years) to take part in research? **Prompt**: adult male, adult women, male greater than 18 years, female greater than 18 years
  - How do you describe a situation where the research participants are required to bring their child to the health facilities for the purpose of the research?
  - How do you describe a situation where the research team visits the research participants’ home repeatedly to give education, to ask several questions, and to play with a child included in the study?
  - Do you think other community members should accompany the research team during home visits? Who do you think is the appropriate member of the community? **Prompt**: HEWs, HDAs,
- Is there anything that we haven’t raised about the decision-making norm in this community?

**Recruitment**

- Why do you think people participate in research?
  - Therapeutic/non-therapeutic distinction – do you think people in the rural area understand they are participating in research, not healthcare?
- Do you think the following issues could affect participant recruitment?
  - Nature of the study (e.g. long follow up, home visit, repeated measurements)
  - Nature of the samples (the inclusion of sick children from the health facilities).
  - Nature of the community (rural, illiterate…etc.)
  - Gender (the involvement of the mother of a child and their decision-making ability to take part in the study)
  - Decision-making norm (individual versus family versus community)
  - Previous exposure to research
  - What other factors affect participant recruitment?
- What problems did you encounter in the past when conducting your research? How did you address them?

**Conclusion**

- Where would we seek guidance if problems occur when conducting our study?
  - **Prompt**: communicate with kebele heads, IRB members, colleagues, refer to national ethics guidelines
- Where would we seek guidance to settle rumors that will arise when conducting our study?
  - **Prompt**: communicate with kebele heads, IRB members, colleagues, refer to national ethics guidelines

# **IDIs guide for kebele heads, community, and religious leaders**

**Personal information**

- Would you please tell me about yourself, including what you do for a living?

**Prompt:** Your role in the community, how long did you stay in the community?

**The community**

- Would you please tell me a bit about the community?

**Prompt**: culture, diversity, language and literacy, poverty, gender structure

**Malnutrition and the intervention**

- How does the community describe malnutrition? **Prompt**: cause, treatment, and prevention?
- How do people react to a malnourished child? In addition, to the family?
- How do you think about the importance of play for the child?

**Prompt**: health, academic achievement, growth, and development?

- How does the community understand the importance of play for the child?

**Prompt**: health, academic achievement, growth, and development?

**Research exposure and consent process**

- Have been involved in a research activity conducted in your Kebele in the past? If yes: What was your role? Why were you consulted? What did you do to help researchers?
- Can you please tell me about your understanding of research?
- Do you understand the difference between treatment and research? Can you please explain to me?
- Do you know that research participants will not get medical care by involving in research works or our study?
- Do you know that participation in research is a voluntary decision?
- Do you know that research participants can decline to participate in the research at any time and you will not be denied healthcare access from health facilities?
  - Do you know that the researchers should give you detail information about the research before participation?
- What information do the research participants like to be provided before making a decision to take part in the research?
- Do you know that the research participants should consent for voluntary participation verbally or through signing on the document (using thumbprint)?
- Are people in this community happy with signing a document/consent form?
- Do the people like to have written information about the study or do they prefer to be told verbally? Why or why not?
- How would the people prefer the information to be presented to you? **Prompt**: individually, in-group with other people, with members of your family…etc.
- What information would like to be told to the participants before deciding to take part in the research?

**Prompt**: What the study is about? Who the researchers are? Why the study is

conducted? Importance of the study? To you, your child? and the community?

Confidentiality and anonymity? Risk and benefit of the study

**Decision-making norm**

- How do people in this community make a decision to take part in research?

**Prompt**: Would the decision be made as a group or would one person make the final decision?

- - - - Do you think a family member should be consulted before an individual is making a decision to take part in research? Prompt: who is the one who should be consulted before making the final decision?
      - Do you think other members of the community should be consulted before making a decision to take part in research? **Prompt**: elderly, religious leaders, kebele leaders.
  - Which member of the community could make an individualized decision to take part in research? **Prompt**: adult male, adult women, male greater than 18 years, female greater than 18 years
  - Who should decide for small children (less than 5 years) to take part in research? **Prompt**: adult male, adult women, male greater than 18 years, female greater than 18 years
  - How do you describe a situation where the research participants are required to bring their child to the health facilities for the purpose of the research?
  - How do you describe a situation where the research team visits the research participants’ home repeatedly to give education, to ask several questions, and to play with a child included in the study?
  - Do you think other community members should accompany the research team during home visits? Who do you think is the appropriate member of the community? **Prompt**: HEWs, HDAs,
- Is there anything that we haven’t raised about the decision-making norm in this community?

**The proposed study**

- Do you think people would participate in this kind of study? Why or why not?
- What are some reasons why individuals won’t participate in research?

**Conclusion**

- Where would we seek guidance if problems occur when conducting our study?
  - **Prompt**: communicate with kebele heads, Community leaders, health workers
- Where would we seek guidance to settle rumors that will arise when conducting our study?
  - **Prompt**: communicate with kebele heads, Community leaders, health workers

# **FGDs guide for community members**

**Malnutrition and the intervention**

- What do you name or the community name the disease, which causes the child too skinny or his/her feet and body to swell?

**Prompt**: cause, treatment, prevention

- How do people react to a child who is too skinny or his/her feet and body are swelling? And to the family?
- How do you think about the importance of play for the child?

**Prompt**: health, academic achievement, growth, and development?

**Research and consent process**

- Have you participated in research in the past?
  - Can you tell me about your experience?
  - How were you approached? What information were you provided? What happened after research etc.?
- Can you please tell me about your understanding of research?
- Have you ever participated in the research? Can you tell me about your experience?
  - **Prompt**: interview, different measurements, information provision (verbal or written), what happened after research?
- Do you understand the difference between treatment and research? Can you please explain to me?
- Do you know that you will not get medical care by involving in research works or our study?
- Do you know that participation in research is your voluntary decision?
- Do you know that you can decline to participate in the research at any time and you will not be denied healthcare access from health facilities?
- Do you know that the researchers should give you detail information about the research before your participation?
- What information do you like to be provided before making a decision to participate in research?
- Do you know that you should consent to your voluntary participation verbally or through signing on the document (using thumbprint)?
- Are you happy with signing a document/ consent form?
- Do you like to have written information about the study or do you prefer to be told verbally? Why or why not?
- How would you prefer the information to be presented to you? **Prompt**: individually, in-group with other people, with members of your family…etc.
- What information would like to be told before you decide to take part in research?

**Prompt**: What the study is about? Who the researchers are? Why the study is

conducted? Importance of the study? To you, your child? and the community?

Confidentiality and anonymity? Risk and benefit of the study

- Do you mind other people knowing about your and your child’s participation in the research?

**Decision-making norm**

- Whom do you prefer to contact with you to ask if you would be interested to participate in research? Why?
- What shall we do to make our discussion fruitful and productive?
- How do you want to express your willingness once you have decided to take part in research? **Prompt**: verbal or written consent
- What do you think about signing a consent form?
- How would you prefer the information to be presented to you? **Prompt**: individually, in-group with other people, with members of your family…etc.
- How would you make a decision to be involved in research? Do you consult your family members before making a decision or do you reach a decision by yourself?
- What information would like to be told before you decide to take part in research?
  - **Prompt**: What the study is about? Who the researchers are? Why the study is conducted? Importance of the study? To you, your child? and the community? Confidentiality and anonymity? Risk and benefit of the study

**Factors affecting recruitment**

- What are some factors affecting participants from taking part in research?
  - - **Prompt**: Not providing information sufficiently, not knowing/understanding the purpose of the study

**The proposed study**

- Do you think people would participate in this kind of study? Why or why not?
- What are some reasons why individuals won’t participate in research?

**Conclusion**

- Where would we seek guidance if problems occur when conducting our study?
  - **Prompt**: communicate with kebele heads, Community leaders, health workers
- Where would we seek guidance to settle rumors that will arise when conducting our study?
  - **Prompt**: communicate with kebele heads, Community leaders, health workers
